# A Comprehensive Review on Serum Ferritin as a Prognostic Marker in Intensive Care Units: Insights Into Ischemic Heart Disease

**DOI:** 10.7759/cureus.57365

**Published:** 2024-03-31

**Authors:** Rushikesh H Dhondge, Sachin Agrawal, Sunil Kumar, Sourya Acharya, Vineet Karwa

**Affiliations:** 1 Medicine, Jawaharlal Nehru Medical College, Datta Meghe Institute of Higher Education and Research, Wardha, IND

**Keywords:** outcome prediction, ischemic heart disease (ihd), critically ill patients, intensive care units (icus), prognostic marker, serum ferritin

## Abstract

Serum ferritin has garnered considerable attention as a prognostic marker in intensive care units (ICUs), offering valuable insights into patient outcomes and clinical management strategies. This comprehensive review examines the role of serum ferritin in predicting outcomes among critically ill patients, with a particular focus on its implications for ischemic heart disease (IHD). Elevated serum ferritin levels have consistently been associated with adverse outcomes in ICU settings, including increased mortality, prolonged hospital stays, and higher morbidity rates. Furthermore, the relationship between serum ferritin levels and IHD underscores its potential as a biomarker for cardiovascular risk assessment in critically ill populations. The review synthesizes existing literature to highlight the predictive value of serum ferritin in assessing illness severity and guiding clinical decision-making in the ICUs. It also explores potential mechanisms linking serum ferritin to adverse outcomes and discusses implications for clinical practice. Integrating serum ferritin measurements into routine assessments could enhance prognostication and risk stratification in ICU patients, while further research is needed to elucidate optimal management strategies and therapeutic targets. Collaborative efforts between clinicians and researchers are essential to advance our understanding of serum ferritin's prognostic value in the ICUs and translate this knowledge into improved patient care and outcomes.

## Introduction and background

In critical care, prognostic markers are crucial in guiding clinical management decisions and predicting patient outcomes. Identifying reliable markers can assist healthcare professionals in risk stratification, early intervention, and optimizing treatment strategies for critically ill patients admitted to intensive care units (ICUs) [1]. Serum ferritin, a marker of body iron stores, has emerged as a potential prognostic indicator in critically ill patients. Elevated serum ferritin levels have been associated with various adverse outcomes, including mortality, morbidity, and prolonged hospital stays, making it a subject of interest in critical care research [2].

Beyond its prognostic value in ICU settings, serum ferritin levels have been linked to ischemic heart disease (IHD), a leading cause of morbidity and mortality worldwide. Understanding the relationship between serum ferritin and IHD is essential for elucidating potential mechanisms underlying the association and exploring novel therapeutic targets [3]. This review aims to comprehensively evaluate the role of serum ferritin as a prognostic marker in ICUs, with a specific focus on its implications for IHD. By synthesizing existing literature, we aim to provide insights into the clinical relevance of serum ferritin levels in predicting outcomes among critically ill patients and shed light on its potential involvement in the pathogenesis of IHD.

## Review

Physiology of serum ferritin

Definition and Structure of Ferritin

Ferritin acts as the cellular storage reservoir for iron, presenting itself as a protein found across diverse life forms, from plants and animals to bacteria and archaea [4]. Structurally, it manifests as a hollow globular protein with an approximate molecular mass of 474 kDa, comprised of 24 subunits capable of housing up to 4500 Fe (III) atoms within an inorganic complex [5]. The formation of ferritin entails the self-assembly of these 24 subunits into a sizable protein shell, adept at encapsulating thousands of iron atoms [6]. Each subunit's configuration encompasses four α-helices (designated as helix A, B, C, and D), which converge to establish the functional entity of ferritin [6]. The protein enclosure exhibits internal and external diameters of roughly 8 nm and 12 nm, respectively, with each subunit contributing to the overall framework of the ferritin molecule [7]. The symmetry of the ferritin cage can adopt either a 432 arrangement (for 24 subunit ferritins) or a 32 configuration (for 12 subunit ferritins), referred to as Dps proteins or mini ferritins, with variations observed in the amino acid sequences across distinct organisms and tissues [8].

Functions of Ferritin in the Body

Iron storage and release: Ferritin is the primary protein for storing iron within the body, preserving it in a nontoxic and soluble form to uphold iron homeostasis [9]. Its crucial function is securely depositing iron within the cells and releasing it when required for essential processes such as hemoglobin synthesis, thereby contributing significantly to the overall iron equilibrium in the body [10].

Iron metabolism: Integral to iron metabolism, ferritin plays a pivotal role in storing and releasing iron according to the body's demands, ensuring a consistent supply of iron for vital physiological functions [11]. The concentration of ferritin in the serum mirrors the total iron stores within the body and is frequently utilized as a diagnostic indicator for conditions like iron deficiency or excess [11].

Protective mechanisms: Iron sequestered within ferritin or hemosiderin within cells offers protection against the detrimental effects of free iron, which can instigate the formation of harmful free radicals from reactive oxygen species (ROS) [12]. The presence of ferritin across various tissue compartments forms a crucial component of vertebrates' protective mechanisms, facilitating the binding and regulation of iron levels effectively [12].

**Figure 1:**
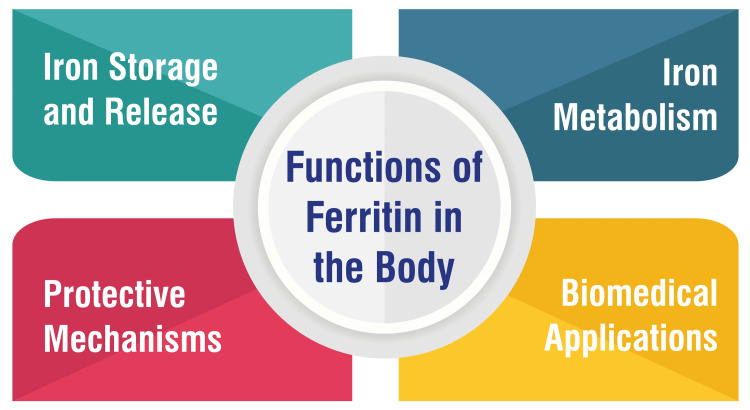
Functions of ferritin in the body The corresponding author Dr. Rushikesh H. Dhondge self-created this figure.

Biomedical applications: Beyond its physiological roles, ferritin finds applications in materials science, serving as a precursor for producing iron nanoparticles utilized across various domains, including carbon nanotube growth and nanoparticle synthesis for biomedical purposes [13]. The protein shells formed by ferritin are harnessed as templates to govern particle growth and prevent aggregation, showcasing its versatility in nanotechnology applications [13]. The functions of ferritin in the body are shown in Figure 1.

Regulation of Serum Ferritin Levels

Regulation at the translation level: The ferritin synthesis is meticulously governed at the translation level of the ferritin gene, with intricate feedback mechanisms orchestrated in response to fluctuations in iron levels [14]. Within ferritin, the H subunit converts excessive intracellular ferric ions into ferrous iron, whereas the L subunit stores ferrous iron within the cells, reflecting the overall ferritin content in the body [14].

Influence of iron status and inflammation: Serum ferritin levels act as indicators of both iron excess and deficiency, with variations mirroring alterations in body iron reserves and inflammatory states [9,14]. Elevated serum ferritin is commonly associated with inflammatory diseases and malignancies, underscoring its significance as a crucial clinical marker [9].

Release mechanism: Despite ongoing research, the precise origin of serum ferritin remains a subject of investigation, with emerging evidence suggesting its derivation from damaged cells rather than active secretion processes in humans [12]. Serum ferritin exhibits immunological relatedness to the L monomer of ferritin, with genetic anomalies in the ferritin L gene contributing to heightened serum levels observed in conditions such as hyperferritinemia [9].

Clinical applications: In clinical practice, serum ferritin is significant in evaluating total body iron stores, diagnosing iron deficiency, and managing conditions associated with iron overload [9]. In neonates, serum ferritin levels are influenced by various factors, including intrauterine growth, maturation, antenatal infections, inflammation, maternal age, and pregnancy-induced hypertension, reflecting its complex interplay with developmental and physiological processes [14].

Methods for assessing serum ferritin

Laboratory Methods for Measuring Serum Ferritin

Immunoturbidimetry method: A widely utilized technique for quantifying ferritin levels is immuno-turbidimetry, commonly employing Roche kits on clinical analyzers such as the Hitachi 912 system [15,16]. This method relies on latex-bound ferritin antibodies that interact with antigens in the sample, forming an antigen-antibody complex, which is subsequently measured turbidimetrically [15]. The degree of turbidity generated is directly proportional to the ferritin concentration, offering a dependable means of assessing ferritin levels in serum or plasma specimens [15].

Sandwich principle method: Another approach for ferritin measurement employs the sandwich principle assay coupled with electrochemiluminescence immunoassay technology, typically conducted on platforms like the cobas® e601 [17]. With a total duration of 18 minutes, this method involves the formation of a sandwich complex comprising a specific antibody and a labeled antibody binding to ferritin within the sample. Subsequently, this complex attaches to solid-phase microparticles, facilitating chemiluminescent emission detection for quantitative analysis [17]. The obtained results are determined using a calibration curve, ensuring precise measurement of ferritin levels in serum samples [17]. These laboratory techniques furnish clinicians and researchers with dependable tools for accurately assessing serum ferritin levels, thereby aiding in diagnosing and managing iron-related conditions and offering invaluable insights into patients' iron status.

Factors Affecting Serum Ferritin Levels

Serum ferritin levels, often assessed to gauge iron reserves within the body, are subject to influence by various factors, including obesity and chronic diseases. Elevated ferritin levels frequently accompany obesity and inflammation [18]. This association highlights the complex interplay between iron metabolism and inflammatory processes, suggesting that ferritin levels may indicate iron status and markers of systemic inflammation in various pathological states. Additionally, the levels of ferritin in the circulation can be affected by fluctuations in hemoglobin values, which are influenced by many factors. These factors include residential elevation above sea level, smoking behavior, environmental conditions, menstrual blood loss in females, maternal care practices, socioeconomic status, and dietary habits [19]. Such variables underscore the intricate web of physiological and environmental factors that can impact the individuals' iron status and serum ferritin levels.

**Figure 2:**
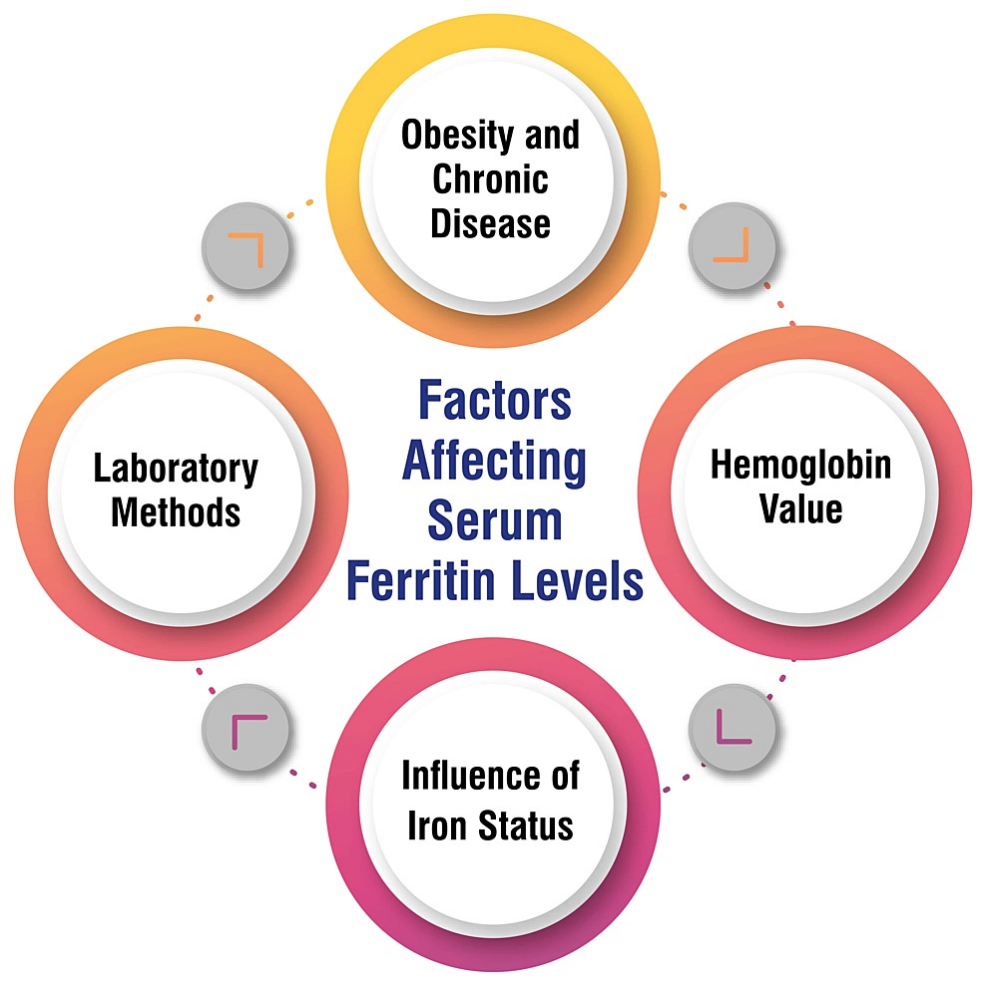
Factors affecting serum ferritin levels The corresponding author Dr. Rushikesh H. Dhondge self-created the figure.

As a crucial marker for assessing iron status, serum ferritin is pivotal in identifying iron deficiency and potential iron overload conditions. Low ferritin levels often signify iron deficiency, while elevated levels may indicate underlying disorders such as hemochromatosis or inflammatory diseases like rheumatoid arthritis or liver ailments [20]. This diagnostic utility underscores the importance of accurately measuring serum ferritin levels in clinical practice to guide appropriate management strategies and interventions for individuals with iron-related disorders. The accuracy and comparability of laboratory methods utilized for measuring ferritin concentrations are paramount in interpreting serum ferritin levels reliably. Various techniques, predominantly based on antigen-antibody reactions, are employed for this purpose, with differences in detection methodologies such as turbidimetry and chemiluminescence [21]. Ensuring consistency and standardization in laboratory methodologies is essential to facilitate accurate interpretation and comparison of serum ferritin data across studies and clinical settings, thus enabling clinicians to make informed decisions regarding patient management and treatment. Factors affecting serum ferritin levels are shown in Figure 2.

Serum ferritin as a prognostic marker in ICU patients

Studies Demonstrating the Prognostic Value of Serum Ferritin in ICU Settings

Research findings have established a robust correlation between serum ferritin levels and patient outcomes within ICU settings, where elevated levels often signal an unfavorable prognosis [22,23]. Observations of serum ferritin dynamics have revealed their capacity to mirror the progression of organ dysfunction and parallel the Sequential Organ Failure Assessment (SOFA) score, thereby offering valuable insights into disease severity and eventual outcomes [22]. Furthermore, serum ferritin has emerged as a reliable predictor of mortality among critically ill patients, furnishing healthcare providers with crucial prognostic information [23,24]. High serum ferritin levels have consistently been associated with prolonged ICU stays, increased requirements for mechanical ventilation, and adverse clinical outcomes, underscoring its predictive utility in assessing patient prognosis [23,24]. The association between elevated serum ferritin levels and indicators of inflammation, tissue damage, and organ dysfunction in critically ill patients underscores its role as a biomarker for disease severity and progression [23,24]. This relationship highlights the importance of monitoring iron metabolism in ICU patients to assess risk and guide management strategies [24]. These studies underscore the significance of serum ferritin assessment as a valuable tool for predicting outcomes, monitoring disease progression, and informing clinical decision-making in managing ICU patients.

Relationship Between Serum Ferritin Levels and Severity of Illness

Patients exhibiting elevated serum ferritin levels tend to experience a heightened incidence of severe illness and liver injury when compared to those with lower levels, as supported by research [25]. Within hospitalized populations, ferritin levels surpassing 2000 ng/ml have demonstrated a significant association with severe diseases, indicating a robust correlation between elevated ferritin levels and disease severity [26]. Serum ferritin has proven effective in discerning the severity of illness, evidenced by a high area under the receiver operating characteristic (ROC) curve, suggesting its potential as a prognostic marker for disease severity [25].

Furthermore, serum ferritin levels have been identified as predictors of outcomes among hospitalized patients, encompassing indicators such as the necessity for ICU admission and mechanical ventilation [23]. Assessments of serum ferritin's predictive capability for severity and mortality have yielded promising outcomes, with ferritin displaying slightly superior predictive value for mortality compared to severity [27]. Studies have unveiled nonlinear relationships between serum ferritin levels and clinical outcomes in conditions like sepsis, where incremental increases in ferritin levels correspond to elevated mortality risks at varying time points [26]. Across a spectrum of disorders, serum ferritin's potential prognostic and diagnostic values have been elucidated, underscoring its significance as a biomarker for evaluating disease severity and forecasting outcomes [26].

Predictive Value of Serum Ferritin for Mortality and Morbidity in ICU Patients

Prognostic significance: Elevated serum ferritin levels in ICU patients with prolonged stays have been consistently linked with adverse outcomes and increased mortality rates [22]. The dynamics of serum ferritin serve as indicators of the progression of organ dysfunction and exhibit a strong correlation with the SOFA score, thereby providing valuable prognostic information regarding patient outcomes [22]. Notably, the predictive accuracy of serum ferritin for patient outcomes rivals that of the SOFA score, underscoring its pivotal role as a prognostic marker in critically ill patients [22].

Clinical implications: Serum ferritin levels are valuable indicators of inflammation, tissue damage, and cellular injury among ICU patients, offering critical insights into disease severity and potential outcomes [3,22]. In conditions such as sepsis, elevated serum ferritin levels have been consistently associated with poorer outcomes, thus highlighting its utility as a biomarker for disease severity and prognosis [3].

Iron metabolism and disease: Abnormal serum ferritin levels extend beyond ICU settings and have been implicated in various diseases, including inflammatory disorders, cardiovascular diseases (CVDs) such as myocardial infarction and coronary artery disease, hepatic disorders, neurological disorders, metabolic disorders, and immune disorders [3,22]. Given this wide-ranging association, monitoring iron metabolism through serum ferritin assessment is critical in risk assessment, disease management, and predicting outcomes among critically ill patients [3].

Mechanisms linking serum ferritin and IHD

Role of Iron Overload in the Pathogenesis of IHD

Excess iron accumulation: Excess iron accumulation, often from heightened gastrointestinal iron absorption or excessive iron administration, poses significant risks to cardiac health. This surplus iron can exacerbate cellular damage and impede cardiac function, particularly in conditions characterized by ischemia and hypoxia [28]. Such iron overload may contribute to the progression of CVDs, exacerbating the pathology and complicating treatment strategies.

Oxidative stress and cellular damage: Elevated iron levels, especially in excess, can generate hydroxyl radicals through oxidative reactions. These radicals, in turn, induce oxidative damage to essential cellular components such as lipids, proteins, and DNA [29]. Of notable concern is the phenomenon of iron-mediated cell death, specifically ferroptosis, which has emerged as a significant contributor to cardiomyocyte damage and is implicated in various cardiovascular disorders, including cardiomyopathy and atherosclerotic CVD [29].

Association with atherosclerosis: Iron overload has been implicated in the pathogenesis of atherosclerosis, with compelling evidence linking elevated serum ferritin concentrations and iron deposition in coronary plaques to an increased risk of myocardial infarction [30]. The catalytically active form of iron within atherosclerotic lesions can generate ROS, thereby fostering oxidative stress and contributing to the initiation and progression of atherogenesis [30]. This association underscores the potential impact of iron metabolism dysregulation on cardiovascular health and disease progression.

Therapeutic implications: Recognizing the deleterious effects of excess iron accumulation on cardiac function, iron chelation therapy has emerged as a promising therapeutic approach. By effectively binding and removing excess iron from the body, iron chelation therapy can potentially ameliorate iron overload-related disorders and attenuate the adverse cardiac effects associated with excess iron accumulation [29].

Clinical significance: Studies have underscored the clinical relevance of assessing ferritin and transferrin saturation levels in predicting prognosis among individuals with coronary artery disease [31]. This emphasizes the importance of monitoring iron metabolism in cardiovascular health, as aberrations in iron status may serve as valuable prognostic indicators and guide therapeutic interventions to optimize patient outcomes [31].

Oxidative Stress and Inflammation Induced by Elevated Serum Ferritin Levels

Inflammatory response and tissue damage: Elevated serum ferritin levels are closely linked to inflammatory responses, which can instigate tissue damage through oxidative stress pathways [32]. Recognized as a significant inflammatory marker, serum ferritin mirrors cellular damage and oxidative stress levels, with its concentrations aligning with biomarkers indicative of hydroxyl radical formation and oxidative stress [12]. Additionally, ferritin plays a pivotal role in modulating the immune response by stimulating the production of anti-inflammatory cytokines and curtailing free radical-induced damage, underscoring its crucial involvement in inflammation and redox biology [33].

Protective role: Ferritin is a protective mechanism during active infections by constraining iron availability to pathogens, thereby bolstering host defenses. This protective role helps curb the production of free radicals and facilitates immunomodulation, thereby aiding in infection containment [33]. Serum ferritin, as a pivotal acute-phase reactant, mirrors the extent of acute and chronic inflammation across various diseases, serving as a valuable indicator for therapeutic interventions to control inflammation in high-risk patients [33].

Iron metabolism and oxidative stress: Dysregulation of iron metabolism, particularly involving ferritin, has been implicated in the pathogenesis of vascular diseases such as diabetes, highlighting the significant impact of iron dysregulation on oxidative stress pathways and disease progression [34]. Notably, in hemodialysis patients, elevated serum ferritin levels are associated with heightened oxidative stress, diminished antioxidant capacity, and an exacerbated inflammatory status, underscoring the pivotal role of ferritin in influencing redox balance and inflammatory responses within this population [35].

Implications of Serum Ferritin in the Development and Progression of IHD

Emerging research underscores a nuanced relationship between serum ferritin levels and CVD and IHD, revealing a U-shaped association wherein optimal levels hover around 60 μg/l. Both elevated and diminished serum ferritin concentrations appear to elevate the risk of CVD and IHD, suggesting a potential threshold effect. Notably, elevated serum ferritin levels are particularly implicated in heightening the risk of coronary artery disease, positioning serum ferritin as a potential biomarker for predicting adverse cardiovascular events [36]. Moreover, in the prognostic realm, heightened serum ferritin levels emerge as indicators of unfavorable outcomes in patients grappling with severe IHD. Patients with elevated serum ferritin levels demonstrate elevated 90-day and one-year mortality rates, marking serum ferritin as a potential prognostic marker in cardiovascular health. Integrating serum ferritin levels into the severity of illness scores has showcased enhancements in the accuracy of predicting patient outcomes, especially in acute myocardial infarction, reflecting its clinical utility in risk stratification and treatment decision-making [3].

Disruptions in iron metabolism, typified by aberrant serum ferritin levels, offer crucial insights into the intricate interplay between iron status and cardiovascular health. Imbalances, whether characterized by low or high serum ferritin levels, have been associated with an augmented risk of heart failure. This highlights the potential role of iron imbalance as a contributing factor to the pathogenesis and progression of heart failure, underscoring the significance of monitoring iron status in CVD management [37]. Furthermore, serum ferritin's clinical relevance extends to its potential as a risk stratifier in critically ill patients grappling with severe IHD. Incorporating serum ferritin levels into the risk assessment protocols holds promise in refining prognostication and tailoring treatment strategies in the intensive care settings. However, amidst accumulating evidence linking serum ferritin levels to CVDs, conflicting findings persist within the literature. While some studies affirm positive associations, others need to establish definitive links, reflecting the complexity and variability inherent in research findings regarding the role of ferritin in CVDs. This underscores the imperative for further investigation to elucidate the precise mechanisms underpinning ferritin's involvement in cardiovascular pathophysiology [3,35,36].

Clinical implications and future directions

Regular monitoring of serum ferritin levels in critically ill patients, especially those afflicted with sepsis or multiple organ dysfunction syndrome (MODS), offers valuable insights into disease severity and prognosis [24,38]. Utilizing ferritin as a prognostic biomarker necessitates the establishment of optimal cut-off values to predict outcomes such as mortality, organ failure, and the necessity for mechanical ventilation, thereby facilitating risk assessment and informing clinical decision-making processes [26,38]. Moreover, the association between elevated serum ferritin levels and adverse outcomes in sepsis and critical illness underscores the imperative for targeted interventions to modulate iron metabolism and mitigate inflammatory responses [26,39]. Therapeutic strategies may encompass iron supplementation approaches for addressing iron deficiency or tailored treatments targeting hyperferritinemia syndromes linked with macrophage activation and systemic inflammation [39].

Furthermore, serum ferritin levels serve as a valuable tool for risk stratification in ICU patients, aiding clinicians in identifying high-risk individuals who may benefit from intensified monitoring, aggressive interventions, or specialized care pathways [24,26]. A comprehensive understanding of the relationship between serum ferritin dynamics and clinical outcomes, including mortality rates, organ failure occurrences, and duration of ICU stays, is pivotal in predicting patient trajectories and optimizing treatment plans [23,24]. Future research endeavors should focus on conducting large-scale prospective studies to validate the prognostic value of serum ferritin in critically ill patients, specifically emphasizing its relevance in conditions like sepsis, MODS, and other critical illnesses [38,39]. Exploring the underlying mechanisms linking elevated serum ferritin levels to adverse outcomes holds promise in identifying potential therapeutic targets and devising novel treatment strategies to enhance patient outcomes in ICU settings [26,39]. By implementing these proactive measures for managing serum ferritin levels in critically ill patients, healthcare providers can elevate risk assessment precision, refine prognosis prediction capabilities, and optimize treatment strategies to improve outcomes and enhance patient care within ICU settings.

## Conclusions

In conclusion, this review has underscored the pivotal role of serum ferritin as a prognostic marker in the ICUs. Elevated serum ferritin levels have consistently emerged as a reliable indicator of adverse outcomes among critically ill patients, including increased mortality, prolonged hospital stays, and heightened morbidity rates. The predictive value of serum ferritin in assessing the severity of illness and guiding clinical management decisions in ICU settings has been well-documented. Moreover, the association between serum ferritin levels and IHD highlights its potential utility as a biomarker for cardiovascular risk assessment in critically ill populations. Integrating serum ferritin measurements into routine assessments could enhance prognostication and risk stratification in ICU patients. Additionally, exploring interventions targeting serum ferritin modulation holds promise for improving outcomes, although further research is warranted to determine optimal management strategies. Future investigations should investigate the underlying mechanisms linking serum ferritin to adverse outcomes in ICU patients and identify potential therapeutic targets to mitigate its impact on morbidity and mortality. Collaborative efforts between clinicians and researchers are crucial for advancing our understanding of serum ferritin's prognostic value in the ICUs and translating this knowledge into tangible improvements in patient care and outcomes.

## Figures and Tables

**Figure 3:**
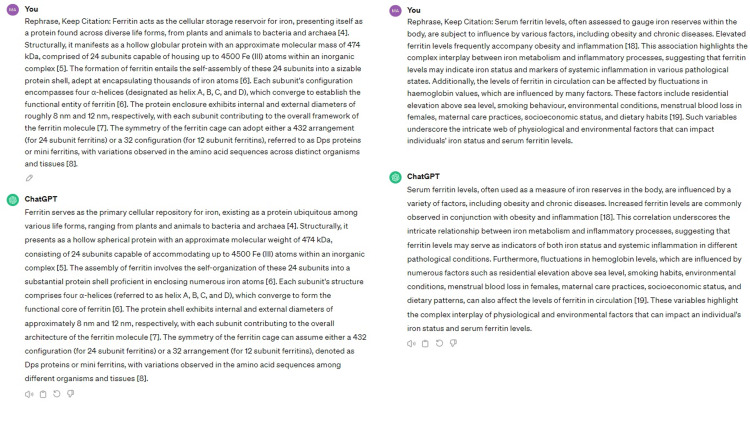
ChatGPT-assisted editing and rephrasing of the review article

## Appendices

The ChatGPT language model (OpenAI, San Francisco, California) was employed to assist in the formulation of key arguments, structuring the content, and refining the language of our manuscript. It provided valuable insights and suggestions throughout the writing process, enhancing the overall coherence and clarity of the article. It was also utilized to assist in editing and rephrasing the work to ensure coherence and clarity in conveying the findings (Figure 3).
